# Schizophrenia Gene Networks and Pathways and Their Applications for Novel Candidate Gene Selection

**DOI:** 10.1371/journal.pone.0011351

**Published:** 2010-06-29

**Authors:** Jingchun Sun, Peilin Jia, Ayman H. Fanous, Edwin van den Oord, Xiangning Chen, Brien P. Riley, Richard L. Amdur, Kenneth S. Kendler, Zhongming Zhao

**Affiliations:** 1 Department of Biomedical Informatics, Vanderbilt University School of Medicine, Nashville, Tennessee, United States of America; 2 Department of Psychiatry, Vanderbilt University School of Medicine, Nashville, Tennessee, United States of America; 3 Washington VA Medical Center, Washington, D. C., United States of America; 4 Virginia Institute for Psychiatric and Behavioral Genetics, Virginia Commonwealth University, Richmond, Virginia, United States of America; 5 Center for Biomarker Research and Personalized Medicine, Virginia Commonwealth University, Richmond, Virginia, United States of America; 6 Department of Human and Molecular Genetics, Virginia Commonwealth University, Richmond, Virginia, United States of America; 7 Department of Cancer Biology, Vanderbilt University Medical Center, Nashville, Tennessee, United States of America; Ohio State University Medical Center, United States of America

## Abstract

**Background:**

Schizophrenia (SZ) is a heritable, complex mental disorder. We have seen limited success in finding causal genes for schizophrenia from numerous conventional studies. Protein interaction network and pathway-based analysis may provide us an alternative and effective approach to investigating the molecular mechanisms of schizophrenia.

**Methodology/Principal Findings:**

We selected a list of schizophrenia candidate genes (SZGenes) using a multi-dimensional evidence-based approach. The global network properties of proteins encoded by these SZGenes were explored in the context of the human protein interactome while local network properties were investigated by comparing SZ-specific and cancer-specific networks that were extracted from the human interactome. Relative to cancer genes, we observed that SZGenes tend to have an intermediate degree and an intermediate efficiency on a perturbation spreading throughout the human interactome. This suggested that schizophrenia might have different pathological mechanisms from cancer even though both are complex diseases. We conducted pathway analysis using Ingenuity System and constructed the first schizophrenia molecular network (SMN) based on protein interaction networks, pathways and literature survey. We identified 24 pathways overrepresented in SZGenes and examined their interactions and crosstalk. We observed that these pathways were related to neurodevelopment, immune system, and retinoic X receptor (RXR). Our examination of SMN revealed that schizophrenia is a dynamic process caused by dysregulation of the multiple pathways. Finally, we applied the network/pathway approach to identify novel candidate genes, some of which could be verified by experiments.

**Conclusions/Significance:**

This study provides the first comprehensive review of the network and pathway characteristics of schizophrenia candidate genes. Our preliminary results suggest that this systems biology approach might prove promising for selection of candidate genes for complex diseases. Our findings have important implications for the molecular mechanisms for schizophrenia and, potentially, other psychiatric disorders.

## Introduction

Schizophrenia (SZ) is a severe mental disorder affecting ∼1% of the population [1]. Family, twin, and adoption studies strongly support that genetic factors play an important role in the etiology of schizophrenia. Recently, numerous genetic studies, including linkage scans and their meta-analyses, candidate gene association analyses, gene expression and genome-wide association studies (GWAS), have identified specific genes/markers and chromosomal regions for the disease [2], [3], [4], [5]. Though these studies present a low replication, more evidence supports that the etiology of schizophrenia involves, rather than single genes/loci with large effect, many genes, each of which contributes a small risk, interacting with each other or with environmental risk factors to cause schizophrenia [3]. In this study we hypothesize that these small effects are organized in networks/pathways and these schizophrenia disease networks/pathways have important features that are distinct from those seen in other diseases such as cancer or Mendelian genetic diseases.

It is increasingly possible to investigate biological networks/pathways of a complex disease at the systems level because of the rapid accumulation of genetic and biological information in the past decade. Recent studies reveal striking correlations between the functions of gene products or gene networks and the features of the diseases they cause [6], [7]. The correlation between the attributes of disease genes is more extensive and stronger than previously thought. This was revealed in an analysis of Gene Ontology (GO) terms and a gene expression pattern of >1,600 genes causing different types of diseases [8]. Further, Goh *et al* [9] found that the majority of disease genes are nonessential and do not encode hub proteins (highly connected proteins) in protein networks. This pattern was different from that previously thought and that observed in cancer genes. Except for only a few diseases such as cancer [10], there has been no systematic investigation of the network properties of a complex disease by examining the whole human interactome. Specifically, we have been unable to find such a detailed examination of features for schizophrenia associated genes.

In this study, we explored network characteristics of 160 schizophrenia candidate genes (SZGenes) that were prioritized by a multi-dimensional evidence-based gene ranking approach [11]. Their global network characteristics and local network environment indicate that SZGenes have their own network properties compared to the cancer genes. We further identified schizophrenia enriched pathways, explored their ability to interact with or influence each other (crosstalk), and constructed the first version of a schizophrenia molecular network (SMN). Finally, we applied our networks/pathways analysis to identify novel candidate genes. Our preliminary experimental verification suggests that this approach might be promising. This study provides useful insights into the molecular mechanisms of schizophrenia at the systems biology level.

## Results

### Global network properties of schizophrenia candidate genes (SZGenes)

To date, no gene has been confirmed to be the cause of schizophrenia. In this study, we used 160 SZGenes that were prioritized based on a multi-dimensional evidence-based gene ranking approach [11]. These genes were selected by integrative evidence from linkage, association, gene expression and literature search. Evaluations from several methods such as independent GWAS *P* values, gene expression features, and GO annotations suggest these genes are useful for follow up bioinformatics analysis (Text S1, Table S1). For comparison, we compiled four other gene sets: cancer genes, essential genes, neurodevelopment-related genes (NeuroGenes) and non-disease, non-essential genes (NDEGenes) (see details in Text S1).

To explore the topological network properties of five gene sets, we first reconstructed a human protein-protein interaction (PPI) network (the human interactome, Text S1). In the human PPI network, nodes represent the proteins encoded by genes and edges (links) represent the interactions identified by experiments. Secondly, we mapped the proteins encoded by the five gene sets onto the whole network, and then calculated the numbers of interactors (namely, degree or connectivity) of nodes, and the shortest-path distances (number of edges from one node to another). They are the basic topological network measures, which provide insights into the architecture of the nodes of interest (i.e., proteins encoded by SZGenes) in the whole network (see Materials and Methods) [12].

#### Moderate connectivity (degree) of SZGenes

**Figure 1:**
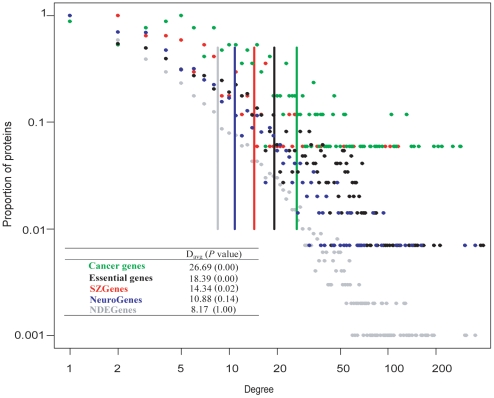
Degree distributions and average degrees (vertical lines) of five gene sets. The Y-axis represents the proportion of proteins having a specific degree. The empirical *P* value in the inserted table indicates whether the observed average degree in a gene set is random from the human interactome.

Figure 1 displays degree distribution and the average degree of the nodes in each gene set. The average degree of SZGenes was 14.34, which was significantly higher than that of NeuroGenes (10.88, Wilcoxon's test, *P* = 0.04) or that of NDEGenes (8.17, *P* = 8.2×10^−8^) but significantly lower than that of essential genes (18.39, *P* = 0.02) or that of cancer genes (26.69, *P* = 6.3×10^−7^). SZGenes had an intermediate connectivity when compared to the four other gene sets, indicating that SZGenes often encode proteins that are moderately connected, rather than highly connected in the human interactome. This observation of higher degree in SZGenes and cancer genes than in NeuroGenes or NDEGenes also supports a recent report that disease genes tend to have higher degrees than non-disease genes [10]. Moreover, proteins encoded by cancer genes, essential genes and SZGenes had significantly more direct interactions than randomly selected nodes (empirical *P* values are 0, 0, 0.02, respectively; here *P* = 0 means no randomly selected node set had a higher average connectivity than the observed values of cancer genes or essential genes), while proteins encoded by NDEGenes had the opposite characteristic (empirical *P* = 1.00, all randomly selected node sets had a higher average degree than the observed values of NDEGenes).

The degree distributions of these five gene sets were strongly right-skewed (Figure 1). Most nodes had low degree while only a portion of nodes had a high degree, which were often defined as “hubs” in network analysis. There are multiple ways to define hubs. Here, we applied the method in Yu *et al* [13] to define hubs. We first plotted the degree distribution of all nodes in the human interactome and then identified the point where the distribution began to straighten out. The point corresponded to degree 13. According to this cutoff, there were 2,165 nodes classified into hubs, which accounted for 20.7% of all nodes in the interactome. This proportion was consistent with the cutoff used in the yeast interactome [14]. The proportion of hub proteins was 29.0% among SZGenes, which was smaller than that of cancer genes (49.4%) or essential genes (38.1%) but larger than that of NeuroGenes (23.9%) or NDEGenes (16.6%). Figure S1 summarizes the detailed distributions of proteins encoded by these gene sets by a degree interval of 3. Among the five gene sets, the proportion of SZGenes was the highest for degree intervals 4–6 and 7–9. For degree intervals 10–12 and 13–17, the proportion of SZGenes was slightly smaller than that of cancer genes and similar to that of essential genes. When we summarized degree intervals 4–17, the proportion of SZGenes was 50.0%, higher than any of the other four gene sets (cancer genes: 47.5%, essential genes: 44.8%, NeuroGenes: 36.6%, and NDEGenes: 38.7%). Thus, the average degree and degree distribution consistently indicate intermediate connectivity of schizophrenia candidate genes.

#### Intermediate shortest-path distance of SZGenes

In a network, shortest-path distance measures how many nodes need to pass through from one node to another [12]. Considering the two nodes might be our interest or not, we calculated shortest-path distance in two ways: characteristic shortest-path distance and global centrality. Characteristic shortest-path distance provides a general view of the relationship between the nodes of interest and all other nodes in the network while global centrality, which calculates the shortest path distance between two nodes belonging to the same gene set of interest, provides a measure of the general view of the interest nodes in the whole network. The detailed distributions and the average distance values of the five genes sets are shown in Figure S2.

Similar to the degree measurement, SZGenes had an intermediate average characteristic shortest-path distance and global centrality among the five gene sets (Figure S2). For example, the average characteristic shortest-path distance of SZGenes was 3.88, greater than that of cancer genes (3.63, Wilcoxon's test, *P* = 5.4×10^−7^) or that of essential genes (3.76, *P* = 0.01) but less than that of NDEGenes (3.98, *P* = 0.001) or NeuroGenes (3.93, *P* = 0.09). The difference in global centrality between SZGenes and the other four gene sets was even stronger than that in characteristic shortest-path distances, e.g., the *P* values by the same test ranged from <2.2×10^−16^ to 2.7×10^−4^. These comparisons indicate that biological signal transferring from one SZGene to another SZGene is faster than that between control genes (e.g. non-disease genes) but slower than that between genes in the essential or cancer gene sets. Therefore, these results imply that perturbation among SZGenes spreads with intermediate efficiency throughout the human protein interactome, which further suggests the damage of gene mutations related to schizophrenia might be weaker than that of mutations in some essential or cancer genes.

### Schizophrenia-specific network

To explore the organization and the environment of the proteins encoded by SZGenes, we extracted the schizophrenia specific subnetwork (SZ-specific network) from the whole network using SZGenes and the Steiner minimal tree algorithm [15]. For comparison purpose, we extracted cancer-specific subnetwork using cancer genes and the same algorithm. The cancer subnetwork is shown in Figure S3.

#### Topological properties of the SZ-specific network

**Figure 2:**
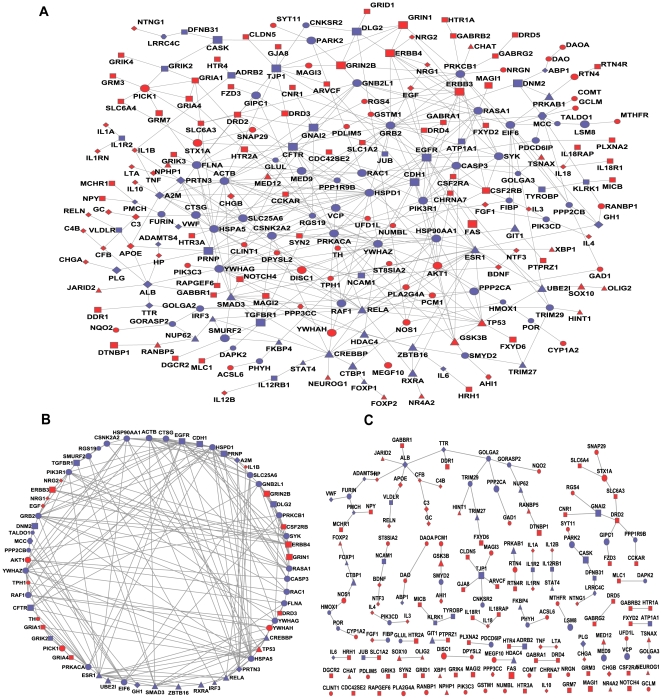
Schizophrenia-specific network. SZGenes are labeled in red and non-SZGenes in blue. Node area corresponds to its degree in the human interactome. Node shape indicates its cellular location: ellipse for cytoplasm, diamond for extracellular space, triangle for nucleus, square for plasma membrane, and hexagon for unknown location. (A) The extracted schizophrenia-specific network from the human interactome. It has 233 nodes and 436 edges. (B) Network for the 63 nodes that could form clusters by CFinder. (C) Networks for the 170 nodes that could not form clusters by CFinder.

The SZ-specific network had 233 nodes and 436 edges (links) while the cancer-specific network had 324 nodes and 844 edges (Figure 2A, Table S2). Among the 160 SZGenes, 135 (75.0%) were included in the SZ-specific network, indicating a high coverage of SZGenes in this subnetwork. However, the coverage was even higher in the cancer-specific network, which included 265 of the 324 cancer genes (94.6%). The average degree of the SZ-specific network (3.74) was lower than that of cancer network (5.21), and consistently, the average shortest-path distance of the SZ-specific network (4.32) was greater than that of the cancer network (3.76) (Table S2). These comparisons revealed that, relative to cancer genes, SZGenes were weakly connected and had a lower efficiency of navigability in the whole interactome. We further tested randomness of the SZ-specific and cancer-specific networks using the Erdos-Renyi model [16] (see Materials and Methods). The test revealed that both gene sets formed non-random networks and had a strong tendency to form clusters, as their shortest paths were significantly different from that of random networks and clustering coefficients were significantly higher than the corresponding random networks (*P* values  = 0, no any random network outperformed the observed SZ- or cancer-specific network).

#### SZGenes distribute peripherally in SZ-specific network

More than half of the genes (135/233 = 57.9%) in the SZ-specific network were SZGenes and they formed 44 direct edges (10.1% of the total 436 edges), indicating SZGenes might approach each other directly or through other nodes to form a small-world network. In a complex network, the ability of nodes to communicate may reflect the network's degree of robustness and error tolerance [17]. Here, we used the *k*-clique clustering method provided by CFinder software [18], which is a popular network analysis method, to examine nodes' distributions. This method identifies the maximally complete subgraphs (*k-*cliques, in which any two nodes have edges) in the networks and the communities, in which two *k*-cliques share exactly *k*-1 nodes. We examined cliques by five *k* values (*k* = 3, 4, 5, 6, 7). When *k* increased, the number of nodes forming clusters decreased and, interestingly, the proportion of SZGenes identified in the protein communities decreased (Table S3). For example, 28.6% of SZGenes formed communities when *k* = 3, but only 14.3% when *k* = 7. This result indicates that SZGenes tend not to appear in the most tightly connected communities. The opposite pattern was observed in cancer genes, which was consistent with a previous report [10].

For *k* = 3, we found 70 cliques involving 63 proteins. These cliques could form a large closely connected subnetwork (Figure 2B). After we removed this subnetwork from the SZ-specific network, connections among the remaining nodes became very loose and nearly corrupt (Figure 2C). Among these 63 proteins, 18 belong to SZGenes, which accounted for 13.3% of the SZGenes in the SZ-specific network. By comparison, we observed that 118 (44.5%) of the 265 cancer genes in the cancer-specific network could form the clusters by 3-clique. The comparison indicates that SZGenes tend to distribute peripherally in the disease specific network rather than reside in the center of clusters, a feature observed in cancer genes [10].

### Schizophrenia enriched pathways

Pathways that are statistically enriched in a set of disease genes may provide important cellular process information for our understanding of the molecular pathology of the disease. We examined schizophrenia enriched pathways (SZ-enriched pathways) using the Fisher's exact test implemented in the Ingenuity Pathway Analysis (IPA) (see Materials and Methods). Further, we explored the interactions and crosstalk between SZ-enriched pathways by taking advantage of both pathway and interaction network data used in this study.

#### Twenty-four significantly enriched pathways for SZGenes

We identified 24 pathways that were significantly enriched for SZGenes (*P* value <0.01) (Table S4). Among these 24 pathways, 9 (37.5%) were directly related to neurodevelopment. This supports the commonly accepted notion of neurodevelopmental abnormalities in schizophrenia [19]. Interestingly, a recent study identified 15 pathways that were overrepresented by genes disrupted in schizophrenia cases versus controls, and these pathways included 5 of our 9 neurodevelopment-related pathways [20]. Four neurotransmitter-related pathways stood out at the top of the list ranked by the significance level: glutamate receptor signaling (ranked 1^st^), serotonin receptor signaling (2^nd^), GABA receptor signaling (5^th^) and dopamine receptor signaling (7^th^). Besides, two pathways, synaptic long-term depression and synaptic long-term potentiation, were in the enriched pathway list. These two pathways are important for synaptic plasticity development and related to schizophrenia [21].

It was worth noting that, among the 24 pathways, 8 were involved in or were related to the immune system. This supports the autoimmune hypothesis of schizophrenia [22]. Recent studies have been accumulating evidence of autoimmune-related genes for the risk of schizophrenia [4], [23]. For example, several interleukin genes (*IL2, IL3*, *IL4*) have been implicated for schizophrenia [23], [24]. Moreover, we found 3 retinoic X receptor (RXR) related signaling pathways: LXR (liver X receptor)/RXR activation, FXR (farnesoid X receptor)/RXR activation, and PPAR (peroxisome proliferators-activated receptor) signaling. RXR acts as a master regulator during ligand-induced transcription activities [25]. Retinoic acid (RA), a metabolic product of retinol, is involved in the development, regeneration and maintenance of the nervous system [21]. The disruption of retinoid has been implicated in the development of schizophrenia [26]. In summary, these enriched pathways suggest that autoimmune and metabolic systems, which may interact with environmental factors, have important roles in the etiology of schizophrenia.

#### Crosstalk among SZ-enriched pathways

Besides searching schizophrenia specific pathways, we took a further step by exploring the interactions and crosstalk between pathways involving in schizophrenia. This analysis assumes that two pathways are likely crosstalk if significantly more proteins or protein interactions are detected between two pathways than expected by chance [27]. There were a total of 276 pathway pairs (links) from the 24 enrich pathways. We found 69 were statistically significantly linked (*P*≤0.01) based on the statistical test described in Materials and Methods.

**Figure 3:**
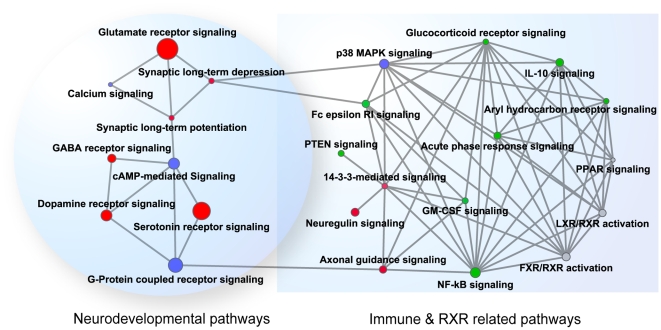
Crosstalk among SZ-enriched pathways. Nodes represent pathways and edges represent crosstalk between pathways. Node area corresponds to its score, which is −10 logarithm of Fisher's exact test *P* value. Red nodes: neurodevelopmental pathways; blue nodes: common signaling pathways; green nodes: immune-related pathways; and grey nodes: RXR (retinoic X receptor) related pathways.

Figure 3 shows the crosstalk of these significantly linked pathways. Two clusters were roughly identified based on the level of interactions between the pathways. One cluster consisted of 9 pathways including 6 neurodevelopment and 3 common signaling pathways, as shown in the left part in Figure 3. The second cluster, as shown in the right part of Figure 3, had 15 pathways, including 1 common signaling pathway, 3 neuronal signaling pathways, 8 immune system-related pathways and 3 RXR-related pathways. The crosstalk among these 15 pathways was much stronger than that in the first cluster. These two clusters were connected by three links. One link, which was between synaptic long-term depression and Fc epsilon RI signaling, appears interesting because it connects the immune-related system to brain development.

### Schizophrenia molecular network (SMN)

**Figure 4:**
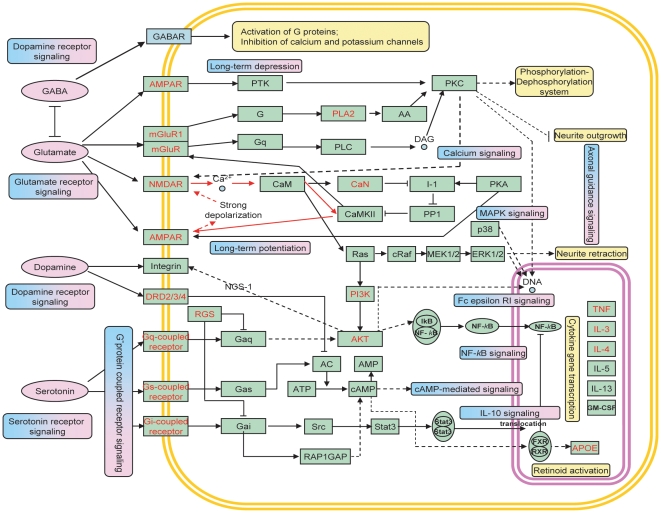
Schizophrenia molecular network (SMN). This network was constructed by using schizophrenia related pathways, protein-protein interactions and literature survey. SZGenes are in red text and neurotransmitters in pink background. The enriched pathways are highlighted in the boxes with a blue and pink background. Several feedback loops are identified including those highlighted with red lines.

To have an overview of the protein-protein interactions and molecular regulations, we constructed a schizophrenia molecular network (SMN). We integrated SZ-enriched pathways with the SZ-specific network, and also enhanced the interactions/regulations by literature surveys. Figure 4 displays the SMN, which includes four neurotransmitters and their transmembrane receptors (neurotransmitter pathways) and their downstream interactions such as activations, inhibitions, and feedback regulations in the cellular system. It also includes many common cellular processes, such as calcium signaling, G-protein coupled receptor signaling, cAMP-mediated signaling, and MAPK signaling pathways. The network also describes the secretion process of small molecules like dopamine; the balance of several effectors, like the inhibition and excitation effects of GABA and glutamate; and the cooperation of regulation via feedback loops after the outer-membrane signal had been transmitted through the membrane into the cellular system.

This network has many feedback loops linking many biological processes. Among those loops, the one from N-methyl-D-aspartate subtype glutamate receptor (NMDAR) to α-amino-3-hydroxy-5-methylisoxazole-4-propionic acid subtype glutamate receptor (AMPAR) appeared to be the shortest. The NMDAR-AMPAR signaling cascade has an important role in apoptosis [28]. Moreover, we observed that those pathways were interlinked via PKC and AKT, suggesting their important roles in the molecular mechanisms of schizophrenia. Both PKC and AKT are protein kinases that are essential in many pathways controlling cell growth and apoptosis. A recent study found a direct link between PKC and AKT [29], which enhanced the crosstalk of the pathways in this molecular network. Overall, this investigation revealed that schizophrenia is a dynamic process caused by dysregulation of multiple pathways that are in crosstalk.

### Application: identification of novel schizophrenia candidate genes

To demonstrate that gene network/pathway analysis is useful in complex diseases, we applied two strategies to select novel candidate genes based on our network/pathway analysis and then performed some experimental verifications.

The first strategy was based on schizophrenia subnetwork analysis. Our SZ-specific network had 233 genes including 135 SZGenes and 98 new genes (non-SZGenes). For each gene in this network, we identified its smallest *P* value using the CATIE [30] and GAIN [31] GWAS markers that mapped to these genes and used the *P* value to represent its significance signal. CATIE and GAIN were the only two publicly available GWAS datasets for schizophrenia when we conducted this study. Mapping SNPs, identification of the smallest *P* value of each gene, and limitation of this approach was described in our previous study [11]. There were 16,892 genes that appeared in both the CATIE and GAIN studies. Among them, 1,745 had the smallest *P* values <0.05 in both the studies. Among the 233 genes in the SZ-specific network, we found 47 whose *P* values were <0.05 in both of the CATIE and GAIN studies. We next tested whether this observation was by chance. We first randomly selected 1,000 sets of 233 genes from the 16,892 common genes. For each random set, we counted the number of genes having *P* values <0.05 in both the CATIE and GAIN datasets (*n*). For these 1,000 sets, we counted the number of sets whose *n* is not less than 47 (*m*). Then, we calculated empirical *P* values by *m*/1,000. We repeated this randomization 10 times to estimate the confidence of this approach. We obtained an empirical *P* = 0, that is, we could not find any random gene set having >47 genes whose *P* values <0.05, as observed in our SZ-specific network. This evaluation suggests that genes in our SZ-network are likely significantly more enriched with small *P* values compared to the overall genome. However, caution should be used in this comparison because this analysis has a bias towards gene length. The SZGenes are overall longer than other human genes, thus, they might have more chance to have small *P* values, assuming GWAS markers were distributed approximately evenly in the human genome.

The evaluation done by using independent GWAS markers indicates that our network analysis may identify a set of new genes with an enriched association signal. Among the 47 genes whose *P* values were <0.05 in both the CATIE and GAIN datasets, 16 were non-SZGenes. We considered these 16 genes as potential candidate genes (Table S5). Most of these genes had not been reported in schizophrenia association studies when we started to collect SZGenes. Next, we extracted 5 subnetworks based on the direct interaction of the 16 non-SZGenes with the nodes encoded by SZGenes and their GWAS *P* values (Figure S4A). This procedure resulted in six potential novel candidate genes (*DLG2*, *EGFR*, *ESR1*, *GRIK2*, *PRKCB1* and *ZBTB16*). Among them, two genes (*EGFR* and *ESR1*) were included for genotyping in our independent project involving 180 genes and other four genes not. Both had markers with *P*<0.05 (unpublished data). Among the 66 SNPs genotyped in *EGFR*, six had *P*<0.05 and the smallest *P* value was 0.003481. Among the 37 SNPs genotyped in *ESR1*, three had *P*<0.05 and the smallest *P* value was 0.001637. Although no marker passed stringent Bonferroni multiple testing and further analysis (e.g., haplotype based analysis) and replication is needed, this preliminary data suggests that our candidate gene selection approach might be effective. Interestingly, for *ESR1*, we did not find any association study reported for schizophrenia when we collected and analyzed data, but we found a recent positive association study [32] during our manuscript preparation. This study further supports our approach.

The second strategy was based on pathway analysis with a combination of network information. Among the 24 SZ-enriched pathways, glutamate receptor signaling pathway ranked at the top. We extracted the glutamate receptor signaling subnetwork from the human interactome using the Steiner minimal tree algorithm. This subnetwork included 18 genes (Figure S4B), 12 of which were among the 160 SZGenes while the other 6 (*DLG2, FLNA*, *GRB2*, *GRIK2*, *HSPA5* and *JUB*) were non-SZGenes. Among them, *GRIK2* has been reported to have positive association with schizophrenia while others have not. GRB2 locates in the center of the subnetwork, which indicates that it might play an important role in the pathway. Among the four well-known neurotransmitter pathways (glutamate receptor signaling, GABA receptor signaling, serotonin receptor signaling and dopamine receptor signaling), GRB2 appeared in all the four pathways and HSPA5 in three pathways, and the interaction between GRB2 and HSPA5 appeared in three pathways. Considering the gene length, functions and chromosome location, we finally selected two genes (*GRB2* and *HSPA5*, both of which are not long) for follow-up experimental verification. Using the Haploview program, we tested the association of seven tagSNPs in gene *GRB2* and two tagSNPs in gene *HSPA5* in our Irish Case-Control Study of Schizophrenia (ICCSS) sample (1,021 cases and 626 controls) [33]. Interestingly, for *GRB2*, five SNPs had *P*<0.05 and the other two SNPs whose *P* values were close to 0.05 (unpublished data). The two smallest *P* values (0.000253 and 0.00313) were significant even after Bonferroni correction. We did not observe any significant signals for the two markers in *HSPA5*. Although more samples and markers are needed for verification, these preliminary results suggest that our network and pathway-based approach for candidate gene selection might be promising.

## Discussion

For complex diseases such as schizophrenia, uncovering susceptibility genes is a challenging but important task. Traditional linkage and association studies have been the primary approaches for this challenge during the last 15 years. Although many loci and genes have been suggested to be linked to schizophrenia, a low replication rate and a lack of functional variants to the risk to schizophrenia have greatly weakened our confidence in the common disease/common variant hypothesis. Furthermore, GWA studies for schizophrenia and other psychiatric disorders have not been as successful as in other diseases or traits such as cancer, body mass, and height [3], [30]. In this study, we hypothesized that the risk for schizophrenia may accumulate among schizophrenia genes that interact through their proteins or in their biological pathways and explored this hypothesis using a systems biology approach. However, this analysis is still preliminary as no genes have been confirmed to be casual in schizophrenia, many genes in the SZGene list may be false positives, and the human interactome and pathway databases are neither complete nor error- or bias-free.

Both schizophrenia and cancer are complex diseases. Our examination of schizophrenia and cancer specific networks revealed major differences, suggesting different effects of genes in causing these two types of diseases. Cancer genes interact more strongly with each other and are more likely to cluster in the network than schizophrenia genes. This feature might be an artifact of data bias: (1) cancer genes might have been studied more by investigators regarding fighting cancers; and (2) although our evaluation suggested SZGenes may be useful, there are likely more false positives in SZGenes than cancer genes and these false positives tend to be non-disease genes [11]. To address the first bias, we examined the properties of the homologous genes in yeast. We downloaded the yeast PPI data from BioGRID [34]. The yeast interaction data is not biased towards cancer or schizophrenia. The same conclusion could be drawn using yeast data. For example, we found a stronger PPI network for cancer homologous genes in yeast. It is worth noting that some genes are associated with both schizophrenia and cancer. It would be interesting to further investigate whether their roles are different in the biological networks/pathways to the diseases.

Despite these limitations, this study provides the first comprehensive view of the network and pathway characteristics of schizophrenia candidate genes. These characteristics demonstrate that schizophrenia is a complex disorder that involves many genes and their interactions. Each of these genes may contribute a small effect to the pathology of schizophrenia. We propose that combing markers/genes at the network or pathway level has greater power to detect an association with schizophrenia than the traditional methods. In this scenario, each mutation might have a different degree of risk effect. Thus, prior information about the mutations in some genes in a network/pathway is helpful for detecting signals in other genes in the same network/pathway. This signal detection is helpful for biomarker discovery and the design of molecular diagnosis. Our preliminary experimental work suggests that this approach is promising.

One recent GWA study indicates that schizophrenia shares substantial polygenetic component with bipolar disorder, another mental disorder [3]. One review of genetic vulnerability to different substances implicates several regions and genes in addiction are also associated with various substances such as alcohol dependence, nicotine, cocaine, opioids, and heroin [35]. The review listed 62 addiction genes [35]. For these genes, we found similar network properties (e.g., a moderate connectivity and intermediate shortest-path distance) and some common neurodevelopment-related pathways that were observed for SZGenes. Overall, the accumulating data and information implicates common genetic components and their interactions in neuropsychiatric disorders.

## Materials and Methods

### Global network properties and significance

In a protein-protein interaction (PPI) network, a node represents a protein and an edge represents an interaction between two nodes. For node *i* in the whole human protein-protein interaction network, we applied two network topological measures to assess the network characteristics of each gene set: (1) degree, the number of links of node *i* in the network [12] and (2) shortest-path length, the number of links of the shortest path traveling from node *i* to another node. The average shortest-path length measures overall navigability of a network. We extended the measurement of shortest-path length in two ways: (1) characteristic shortest-path distance, which was the shortest-path distance from a gene to another gene in the whole network, and (2) global centrality, which was the shortest-path length between two proteins both belonging to the same gene list, allowing transitions through proteins in other categories.

To test the significance of the network properties of a gene set, we developed an empirical re-sampling approach. For each gene set of interest having *n* genes, we randomly selected *n* genes from all available proteins (i.e., random gene set) and calculated their average degree and characteristic shortest-path distance. We repeated this re-sampling 1,000 times. To estimate the significance of average degree observed in the gene set of interest, we counted the number (*N_i_*) of random gene sets whose average degree was higher than the observed average degree and then calculated an empirical *P* value  = *N_i_*/1,000. Similarly, for shortest-path distance, we counted the number (*M_i_*) of random gene sets whose average shortest-path distance is smaller than the observed distance and then calculated an empirical *P* value  = *M_i_*/1,000.

### Construction of SZ-specific network

To extract a network for SZGenes, we first reconstructed a PPI network (the human interactome, Text S1). Among the 160 SZGenes, 137 were mapped into the human interactome. We extracted the subnetwork as a SZ-specific network from the whole human interactome by using Steiner minimal tree algorithm [15]. In this algorithm, the subnetwork starts with an interesting protein set and expands step-by-step until all interesting proteins are netted. Then, the network is simplified to a minimum net containing SZGenes by shortest-path lengths among interest proteins. To test the non-randomness of the subnetwork, we first generated 1,000 random networks with the same number of nodes and links in the SZ-specific network. We applied the Erdos-Renyi model in the R igraph package to the randomization process. Then we estimated the significance of non-randomness by examining network measures such as average degree, average shortest-path distance and clustering coefficient. The empirical *P* values were calculated similarly as in the subsection above. For comparison, we obtained a cancer-specific network and estimated the significance of its non-randomness by running 1,000 random networks, like we did for the SZ-specific network.

### SZ-enriched pathways and their crosstalk

We searched the pathways of 160 SZGenes in the Ingenuity System (http://www.ingenuity.com) and found 101 canonical pathways. We further applied the following two criteria to identify SZ-enriched pathways: 1) the score, which is -10 logarithm of Fisher's exact test *P* value, in a pathway is >2; and 2) the number of SZGenes involved in a pathway is >5. For pathway crosstalk, we considered both common proteins (nodes) and common interactions (edges) between any two pathways. For any pair of SZ-enriched pathways, we calculated a 2×2 contingency table, which includes four counts: *n*, *N*-*n*, *r*, *R*-*r* where *n* is the number of common nodes (or links) between two tested pathways in the pair; *N* is the number of total nodes (or links) of the two tested pathways, *r* is the average number of common nodes (or links) between all possible pairs of SZ-enriched pathways and *R* is the average number of proteins (or links) of all possible pairs of SZ-enriched pathways. For nodes (or links), we used Fisher's exact test to calculate *P* values and adjusted them by false discovery rate (FDR) using Benjamini-Hochberg procedure [36]. Therefore, for each pair of the pathways, we calculated two *P* values (*P_nodes_* and *P_links_*). We choose the smaller *P* value as the criteria of pathway crosstalk. If the smaller *P* value is less than 0.01, we regarded the two pathways significantly have crosstalk.

## Supporting Information

Text S1 Detailed Materials and Methods. In this Supporting Information Text S1, we include additional technical information.
(0.06 MB DOC)

Table S1 GO terms significantly enriched in SZGenes (schizophrenia genes) compared to NDEGenes (non-disease non-essential genes).
(0.10 MB DOC)

Table S2 Comparison of genes distributed in SZ-specific network with those in cancer-specific network.
(0.04 MB DOC)

Table S3 Comparison of the number of nodes forming clusters by different K-cliques in schizophrenia and cancer gene subnetworks.
(0.03 MB DOC)

Table S4 Pathways significantly enriched for schizophrenia candidate genes.
(0.05 MB DOC)

Table S5 Information of 16 genes in association studies and GWA studies.
(0.05 MB DOC)

Figure S1 Degree distribution of five gene sets. Y-axis represents the proportion of proteins having a specific degree.
(0.31 MB TIF)

Figure S2 Shortest-path distance of five gene sets. (A) Characteristic shortest-path distance distribution. Y-axis is the proportion of proteins having a specific characteristic shortest-path distance. The average characteristic shortest-path distance and its empirical P value for randomness from the human interactome are shown in the table inside. (B) Global centrality distribution. Y-axis is the proportion of proteins having a specific global centrality. Vertical line represents the average global centrality of each gene set, which is also summarized in the inside of the figure.
(0.92 MB TIF)

Figure S3 Cancer-specific network. Cancer genes are labeled in red and non-cancer genes in blue. Node area corresponds to its degree in the human interactome. Node shape indicates its cellular location: ellipse for cytoplasm, diamond for extracellular space, triangle for nucleus, square for plasma membrane, and hexagon for unknown location.
(0.67 MB TIF)

Figure S4 Selection of novel schizophrenia candidate genes. Nodes in red denote SZGenes and nodes in grey and green denote non-SZGenes. (A) Direct interactors of five potential schizophrenia candidate genes (in green), which are non-SZGenes but had P values <0.05 in both GAIN and CATIE GWA studies. In these subnetworks, the nodes whose genes having P value <0.05 in GAIN are labeled in red asterisk and having P value <0.05 in CATIE are labeled in blue asterisk. (B) An extracted glutamate receptor signaling subnetwork.
(0.35 MB TIF)
