# Supplementary material for: Schizophrenia Gene Networks and Pathways and Their Applications for Novel Candidate Gene Selection
Source: PLoS One. 2010 Jun 29;5(6):e11351. doi: 10.1371/journal.pone.0011351 (PMC2894047; doi:10.1371/journal.pone.0011351)
Supplement: Text S1 — Detailed Materials and Methods. In this Supporting Information Text S1, we include additional technical information. (0.06 MB DOC) [file pone.0011351.s001.doc]

### **Text S1. Detailed Materials and Methods**

### GO evaluation of schizophrenia candidate genes (SZGenes)

Candidate genes for schizophrenia (SZGenes) were selected from a list of 160 genes that were prioritized based on multi-dimensional evidence, including association, linkage, gene expression and literature search [1].

These genes have been successfully evaluated by two independent schizophrenia GWAS datasets and gene expression in human tissues [1]. Here we further evaluated whether these genes share similar gene function annotations [2], especially those related to neurodevelopment, as referred to as “the usual suspects” for schizophrenia [3]. We examined the enriched Gene Ontology (GO) terms of these 160 SZGenes. Fifty GO terms were significantly associated with at least 6 SZGenes and their hierarchical levels in GO tree were at least 4 (Table S1). Overall, these terms tend to be related to signal transduction, neurodevelopmental processes and apoptosis. Among them, 12 (24%) terms are directly related to neurodevelopment. They were synaptic transmission, transmission of nerve impulse, nervous system development, regulation of neurotransmitter levels, neurological system process, neuron development, neurotransmitter receptor activity, glutamate receptor activity, ionotropic glutamate receptor activity, extracellular-glutamate-gated ion channel activity, GABA receptor activity, and postsynaptic membrane. Seven of these twelve terms were among the top 10 GO terms in each GO category. These results further confirmed that this gene set is useful for follow up bioinformatics analysis.

### Construction of the human interactome

There are several public databases including human protein-protein interaction (PPI) data. To construct the reliable and “complete” human interactome, we retrieved human PPI data from six major databases of protein interactions with experimental evidence: Human Protein Reference Database (HPRD, Release 7) [4], BIND (20060525) [5], IntAct [6], MINT [7], Reactome (version 24) [8], and DIP (version Hsapi20070707) [9]. After excluding the redundant and self interacting pairs, we had 53,255 pairs of proteins encoded by 10,459 genes. These protein pairs were considered as an approximation of a full set of human protein-protein interactions or the human interactome.

### Compilation of gene sets for comparison

**Human cancer genes.** We extracted 291 cancer genes from a comprehensive census of human cancer genes [10], in which 280 genes could be found in the gene information file from the NCBI Gene database (<ftp://ftp.ncbi.nlm.nih.gov/gene/>) (May, 14, 2008). We used these 280 genes as cancer associated genes. Among them, 265 genes could be mapped onto the human interactome.

**Human disease genes.** Disease genes were obtained from the OMIM (Online Mendelian Inheritance in Man) database (<http://www.ncbi.nlm.nih.gov/entrez/query.fcgi?db=OMIM>) [4]. There were 4,850 records in the morbidmap file (May 5, 2008). We restricted the entries with at least 3 evidences. This restriction resulted in a list of 2,168 genes with unique OMIM IDs.

**Human essential genes.** We used the method in Goh *et al* [11] to obtain essential genes. We retrieved the human-mouse orthologs and mouse phenotype data from the Mouse Genome Informatics [12] on September 5, 2008. There were 2,217 mouse-lethal human orthologs, of which 708 have known human disease associations (31.9%). Among them, 1,896 genes could be mapped onto the human interactome.

**Human neurodevelopment-related genes.** We obtained neurodevelopment genes by searching genes with appropriate GO annotations. First, we compiled 17 neurodevelopment related keywords, which is listed in the Schizophrenia Gene Resource (SZGR) (<http://bioinfo.mc.vanderbilt.edu/SZGR/html/godoc.jsp>). Second, we mapped these keywords to GO term descriptions, and extracted their GO IDs. The GO term descriptions were downloaded from the GO website (<http://www.geneontology.org/ontology/>). Third, we mapped these GO IDs to the human gene GO annotations and identified 1,577 genes. Thus, each of these 1,577 genes has at least one neurodevelopment related GO annotation. Finally, after removing the human disease genes, we had 1,221 genes. These genes were considered as the non-disease neurodevelopment genes (abbreviated as NeuroGenes). Among them, 870 genes could be mapped in the human interactome.

**Human non-disease, non-essential genes (NDEGenes).** For control purpose, we extracted 25,598 protein-coding genes from the NCBI Gene database. After excluding SZGenes, cancer genes, essential genes and disease genes, we had 21,799 genes. We considered them as human non-disease, non-essential genes (NDEGenes). Among them, 7,122 genes could be mapped in the human interactome.

### References

1. Sun J, Jia P, Fanous AH, Webb BT, van den Oord EJ, et al. (2009) A multi-dimensional evidence-based candidate gene prioritization approach for complex diseases-schizophrenia as a case. Bioinformatics 25: 2595-6602.

2. Aerts S, Lambrechts D, Maity S, Van Loo P, Coessens B, et al. (2006) Gene prioritization through genomic data fusion. Nat Biotechnol 24: 537-544.

3. Ross CA, Margolis RL, Reading SA, Pletnikov M, Coyle JT (2006) Neurobiology of schizophrenia. Neuron 52: 139-153.

4. Peri S, Navarro JD, Amanchy R, Kristiansen TZ, Jonnalagadda CK, et al. (2003) Development of human protein reference database as an initial platform for approaching systems biology in humans. Genome Res 13: 2363-2371.

5. Bader GD, Donaldson I, Wolting C, Ouellette BF, Pawson T, et al. (2001) BIND--The Biomolecular Interaction Network Database. Nucleic Acids Res 29: 242-245.

6. Hermjakob H, Montecchi-Palazzi L, Lewington C, Mudali S, Kerrien S, et al. (2004) IntAct: an open source molecular interaction database. Nucleic Acids Res 32: D452-455.

7. Chatr-aryamontri A, Ceol A, Palazzi LM, Nardelli G, Schneider MV, et al. (2007) MINT: the Molecular INTeraction database. Nucleic Acids Res 35: D572-574.

8. Joshi-Tope G, Gillespie M, Vastrik I, D'Eustachio P, Schmidt E, et al. (2005) Reactome: a knowledgebase of biological pathways. Nucleic Acids Res 33: D428-432.

9. Salwinski L, Miller CS, Smith AJ, Pettit FK, Bowie JU, et al. (2004) The Database of Interacting Proteins: 2004 update. Nucleic Acids Res 32: D449-451.

10. Futreal PA, Coin L, Marshall M, Down T, Hubbard T, et al. (2004) A census of human cancer genes. Nat Rev Cancer 4: 177-183.

11. Goh KI, Cusick ME, Valle D, Childs B, Vidal M, et al. (2007) The human disease network. Proc Natl Acad Sci USA 104: 8685-8690.

12. Blake JA, Eppig JT, Bult CJ, Kadin JA, Richardson JE (2006) The Mouse Genome Database (MGD): updates and enhancements. Nucleic Acids Res 34: D562-567.
