# Supplementary material for: Schizophrenia Gene Networks and Pathways and Their Applications for Novel Candidate Gene Selection
Source: PLoS One. 2010 Jun 29;5(6):e11351. doi: 10.1371/journal.pone.0011351 (PMC2894047; doi:10.1371/journal.pone.0011351)
Supplement: Table S1 — GO terms significantly enriched in SZGenes (schizophrenia genes) compared to NDEGenes (non-disease non-essential genes). (0.10 MB DOC) [file pone.0011351.s002.doc]

**Table S1** GO terms significantly enriched in SZGenes (schizophrenia genes) compared to NDEGenes (non-disease non-essential genes)

| GO code | | GO term description | Number of genes | *P* value ^a^ |
| --- | --- | --- | --- | --- |
| **Biological process: 27** | | | | |
|  | GO:0007267 | Cell-cell signaling | 39 | 7.42 × 10^-22^ |
|  | GO:0007268 | Synaptic transmission | 28 | 1.98 × 10^-21^ |
|  | GO:0019226 | Transmission of nerve impulse | 28 | 1.08 × 10^-20^ |
|  | GO:0007399 | Nervous system development | 21 | 1.92 × 10^-9^ |
|  | GO:0048731 | System development | 21 | 2.07 × 10^-9^ |
|  | GO:0001505 | Regulation of neurotransmitter levels | 7 | 5.20 × 10^-8^ |
|  | GO:0050877 | Neurological system process | 31 | 5.38 × 10^-8^ |
|  | GO:0007166 | Cell surface receptor linked signal transduction | 43 | 6.36 × 10^-8^ |
|  | GO:0007165 | Signal transduction | 66 | 1.94 × 10^-7^ |
|  | GO:0043066 | Negative regulation of apoptosis | 11 | 3.18 × 10^-7^ |
|  | GO:0043069 | Negative regulation of programmed cell death | 11 | 4.05 × 10^-7^ |
|  | GO:0042981 | Regulation of apoptosis | 16 | 4.67 × 10^-7^ |
|  | GO:0043067 | Regulation of programmed cell death | 16 | 5.73 × 10^-7^ |
|  | GO:0007187 | G-protein signaling, coupled to cyclic nucleotide second messenger | 9 | 1.93 × 10^-6^ |
|  | GO:0019932 | Second-messenger-mediated signaling | 12 | 2.85 × 10^-6^ |
|  | GO:0019935 | Cyclic-nucleotide-mediated signaling | 9 | 3.24 × 10^-6^ |
|  | GO:0006916 | Anti-apoptosis | 9 | 8.29 × 10^-6^ |
|  | GO:0006807 | Nitrogen compound metabolic process | 14 | 8.98 × 10^-6^ |
|  | GO:0006915 | Apoptosis | 18 | 2.12 × 10^-5^ |
|  | GO:0048518 | Positive regulation of physiological process | 17 | 2.20 × 10^-5^ |
|  | GO:0012501 | Programmed cell death | 18 | 2.40 × 10^-5^ |
|  | GO:0006811 | Ion transport | 21 | 3.22 × 10^-5^ |
|  | GO:0008219 | Cell death | 18 | 4.03 × 10^-5^ |
|  | GO:0048666 | Neuron development | 6 | 4.65 × 10^-5^ |
|  | GO:0043086 | Negative regulation of enzyme activity | 6 | 5.44 × 10^-5^ |
|  | GO:0019933 | cAMP-mediated signaling | 6 | 7.35 × 10^-5^ |
|  | GO:0007188 | G-protein signaling, coupled to cAMP nucleotide second messenger | 6 | 7.35 × 10^-5^ |
| **Molecular function: 15** | | | | |
|  | GO:0005230 | Extracellular ligand-gated ion channel activity | 15 | 1.30 × 10^-16^ |
|  | GO:0015276 | Ligand-gated ion channel activity | 15 | 2.71 × 10^-14^ |
|  | GO:0030594 | Neurotransmitter receptor activity | 14 | 1.08 × 10^-11^ |
|  | GO:0008227 | Amine receptor activity | 10 | 2.13 × 10^-11^ |
|  | GO:0004888 | Transmembrane receptor activity | 43 | 3.91 × 10^-11^ |
|  | GO:0008066 | Glutamate receptor activity | 10 | 1.30 × 10^-10^ |
|  | GO:0005231 | Excitatory extracellular ligand-Gated ion channel activity | 9 | 2.16 × 10^-10^ |
|  | GO:0008083 | Growth factor activity | 14 | 3.41 × 10^-10^ |
|  | GO:0004970 | Ionotropic glutamate receptor activity | 7 | 1.96 × 10^-9^ |
|  | GO:0005234 | Extracellular-glutamate-gated ion channel activity | 7 | 1.96 × 10^-9^ |
|  | GO:0015267 | Alpha-type channel activity | 20 | 2.53 × 10^-9^ |
|  | GO:0005216 | Ion channel activity | 19 | 8.07 × 10^-9^ |
|  | GO:0005102 | Receptor binding | 23 | 1.97 × 10^-7^ |
|  | GO:0016917 | GABA receptor activity | 6 | 6.71 × 10^-7^ |
|  | GO:0015075 | Ion transmembrane transporter activity | 22 | 1.74 × 10^-6^ |
| **Cellular component: 8** | | | | |
|  | GO:0045211 | Postsynaptic membrane | 17 | 4.45 × 10^-18^ |
|  | GO:0005886 | Plasma membrane | 55 | 4.92 × 10^-15^ |
|  | GO:0031226 | Intrinsic to plasma membrane | 41 | 6.10 × 10^-12^ |
|  | GO:0044459 | Plasma membrane part | 45 | 6.99 × 10^-12^ |
|  | GO:0005887 | Integral to plasma membrane | 40 | 1.95 × 10^-11^ |
|  | GO:0005615 | Extracellular space | 18 | 1.30 × 10^-6^ |
|  | GO:0016020 | Membrane | 86 | 3.24 × 10^-5^ |
|  | GO:0044425 | Membrane part | 75 | 6.20 × 10^-5^ |

^a^ *P* values were calculated by Fisher's exact test between schizophrenia candidate genes (SZGenes) and non-disease, non-essential genes (NDEGenes).
