# Supplementary material for: Schizophrenia Gene Networks and Pathways and Their Applications for Novel Candidate Gene Selection
Source: PLoS One. 2010 Jun 29;5(6):e11351. doi: 10.1371/journal.pone.0011351 (PMC2894047; doi:10.1371/journal.pone.0011351)
Supplement: Table S2 — Comparison of genes distributed in SZ-specific network with those in cancer-specific network. (0.04 MB DOC) [file pone.0011351.s003.doc]

**Table S2** Comparison of genes distributed in SZ-specific network with those in cancer-specific network

|  | SZ network | Cancer network | *P* value (χ^2^ test) |
| --- | --- | --- | --- |
| Network property |  |  |  |
| Average degree | 3.74 | 5.21 |  |
| Average shortest-path distance | 4.32 | 3.76 |  |
| Clustering coefficient | 0.16 | 0.14 |  |
| Genes |  |  |  |
| Total genes | 233 | 324 |  |
| Disease-specific genes | 135 (57.9%) | 265 (81.8%) | 1.2 × 10^-9^ |
| Non-disease-specific genes | 98 | 59 |  |
| Number of disease genes with direct edge | 56 | 192 | 3.1 × 10^-9^ |
| Number of disease genes with indirect edge | 79 | 73 |  |
| Links |  |  |  |
| Total edges | 436 | 844 |  |
| Direct edges | 44 (10.1%) | 395 (46.8%) | 6.4 × 10^-39^ |
| Indirect edges | 392 | 449 |  |
| Cluster (3-cliques) |  |  |  |
| Number of disease genes forming clusters | 18 (13.3%) | 118 (44.5%) | 9.6 × 10^-10^ |
| Number of disease genes not forming clusters | 117 | 147 |  |
