# Supplementary material for: Schizophrenia Gene Networks and Pathways and Their Applications for Novel Candidate Gene Selection
Source: PLoS One. 2010 Jun 29;5(6):e11351. doi: 10.1371/journal.pone.0011351 (PMC2894047; doi:10.1371/journal.pone.0011351)
Supplement: Table S3 — Comparison of the number of nodes forming clusters by different K-cliques in schizophrenia and cancer gene subnetworks. (0.03 MB DOC) [file pone.0011351.s004.doc]

**Table S3** Comparison of the number of nodes forming clusters by different *K*-cliques in schizophrenia and cancer gene subnetworks

| *k*-clique | SZGenes | | Cancer genes | |
| --- | --- | --- | --- | --- |
|  | Number of nodes forming clusters | SZGenes (%) | Number of nodes forming clusters | Cancer genes (%) |
| 3 | 63 | 18 (**28.6**) | 154 | 118 (**76.6**) |
| 4 | 27 | 5 (**18.5**) | 62 | 48 (**77.4**) |
| 5 | 14 | 2 (**14.3**) | 32 | 27 (**84.4**) |
| 6 | 14 | 2 (**14.3**) | - | - |
| 7 | 7 | 0 | - | - |
