# Supplementary material for: Schizophrenia Gene Networks and Pathways and Their Applications for Novel Candidate Gene Selection
Source: PLoS One. 2010 Jun 29;5(6):e11351. doi: 10.1371/journal.pone.0011351 (PMC2894047; doi:10.1371/journal.pone.0011351)
Supplement: Table S4 — Pathways significantly enriched for schizophrenia candidate genes. (0.05 MB DOC) [file pone.0011351.s005.doc]

**Table S4** Pathways significantly enriched for schizophrenia candidate genes

| Ranking | Pathway | Score ^a^ |
| --- | --- | --- |
| 1 | Glutamate receptor signaling ^b, f^ | 9.89 |
| 2 | Serotonin receptor signaling ^b^ | 8.04 |
| 3 | G-protein coupled receptor signaling ^c, f^ | 6.60 |
| 4 | GABA receptor signaling ^b^ | 5.56 |
| 5 | cAMP-mediated signaling ^c^ | 5.19 |
| 6 | Dopamine receptor signaling ^b^ | 5.16 |
| 7 | NF-*k*B signaling ^d^ | 4.69 |
| 8 | p38 MAPK signaling ^c^ | 4.44 |
| 9 | FXR/RXR activation ^e^ | 3.85 |
| 10 | Neuregulin signaling ^b, f^ | 3.82 |
| 11 | IL-10 signaling ^d^ | 3.72 |
| 12 | Fc epsilon RI signaling ^d^ | 3.55 |
| 13 | Axonal guidance signaling ^b, f^ | 3.52 |
| 14 | LXR/RXR activation ^e^ | 3.50 |
| 15 | Acute phase response signaling ^d^ | 3.39 |
| 16 | GM-CSF signaling ^d^ | 2.94 |
| 17 | PTEN signaling ^d^ | 2.94 |
| 18 | 14-3-3-mediated signaling ^b^ | 2.80 |
| 19 | Glucocorticoid receptor signaling ^d^ | 2.73 |
| 20 | Synaptic long-term depression ^b, f^ | 2.61 |
| 21 | Aryl hydrocarbon receptor signaling ^d^ | 2.61 |
| 22 | Synaptic long-term potentiation ^b, f^ | 2.56 |
| 23 | PPAR signaling ^e^ | 2.16 |
| 24 | Calcium signaling ^c^ | 2.09 |

^a^ Score = -log_10_(*P* value), where *P* value was calculated by Fisher’s exact test.

^b^ Pathways directly related to neurodevelopment.

^c^ Pathways related to signal transduction.

^d^ Pathways involved in or related to immune system.

^e^ Retinoic X receptor (RXR) related pathways.

^f^ Pathways evaluated by genes disrupted in schizophrenia cases versus controls in Walsh et al [1].

**Reference**

1. Walsh T, McClellan JM, McCarthy SE, Addington AM, Pierce SB, et al. (2008) Rare structural variants disrupt multiple genes in neurodevelopmental pathways in schizophrenia. Science 320: 539-543.
