# Supplementary material for: Schizophrenia Gene Networks and Pathways and Their Applications for Novel Candidate Gene Selection
Source: PLoS One. 2010 Jun 29;5(6):e11351. doi: 10.1371/journal.pone.0011351 (PMC2894047; doi:10.1371/journal.pone.0011351)
Supplement: Table S5 — Information of 16 genes in association studies and GWA studies. (0.05 MB DOC) [file pone.0011351.s006.doc]

**Table S5** Information of 16 genes in association studies and GWA studies

| Gene | Association studies | | GWAS *P* value | |
| --- | --- | --- | --- | --- |
|  | Number of positive studies ^a^ | Number of negative studies ^b^ | CATIE | GAIN |
| *DFNB31* | NA ^c^ | NA | 0.0032 | 0.0050 |
| *DLG2* | NA | NA | 0.0007 | 0.0049 |
| *EGFR* | 1 | 1 | 0.0222 | 0.0125 |
| *ESR1* | NA | 2 | 0.0413 | 0.0102 |
| *FOXP1* | NA | NA | 0.0000094 | 0.0205 |
| *GRIK2* | 1 | 3 | 0.0169 | 0.0039 |
| *HDAC4* | NA | NA | 0.0037 | 0.0095 |
| *IL1R2* | NA | NA | 0.0120 | 0.0099 |
| *LRRC4C* | NA | NA | 0.0070 | 0.0020 |
| *MCC* | NA | NA | 0.0034 | 0.0002 |
| *NCAM1* | NA | 2 | 0.0133 | 0.0023 |
| *PARK2* | NA | NA | 0.0032 | 0.0038 |
| *PRKCB1* | NA | NA | 0.0208 | 0.0125 |
| *SMYD2* | NA | NA | 0.0078 | 0.0312 |
| *VWF* | NA | NA | 0.0297 | 0.0139 |
| *ZBTB16* | NA | NA | 0.0194 | 0.0075 |

^a^ Positive: at least one study had significant (*P* <0.05) association based on the data downloaded in April, 2008 in SchizophreniaGene database (<http://www.schizophreniaforum.org/res/sczgene/>).

^b^ Negative: no evidence for significant association.

^c^ NA = not available
