# Supplementary figures and images for: Schizophrenia Gene Networks and Pathways and Their Applications for Novel Candidate Gene Selection
Source: PLoS One. 2010 Jun 29;5(6):e11351. doi: 10.1371/journal.pone.0011351 (PMC2894047; doi:10.1371/journal.pone.0011351)

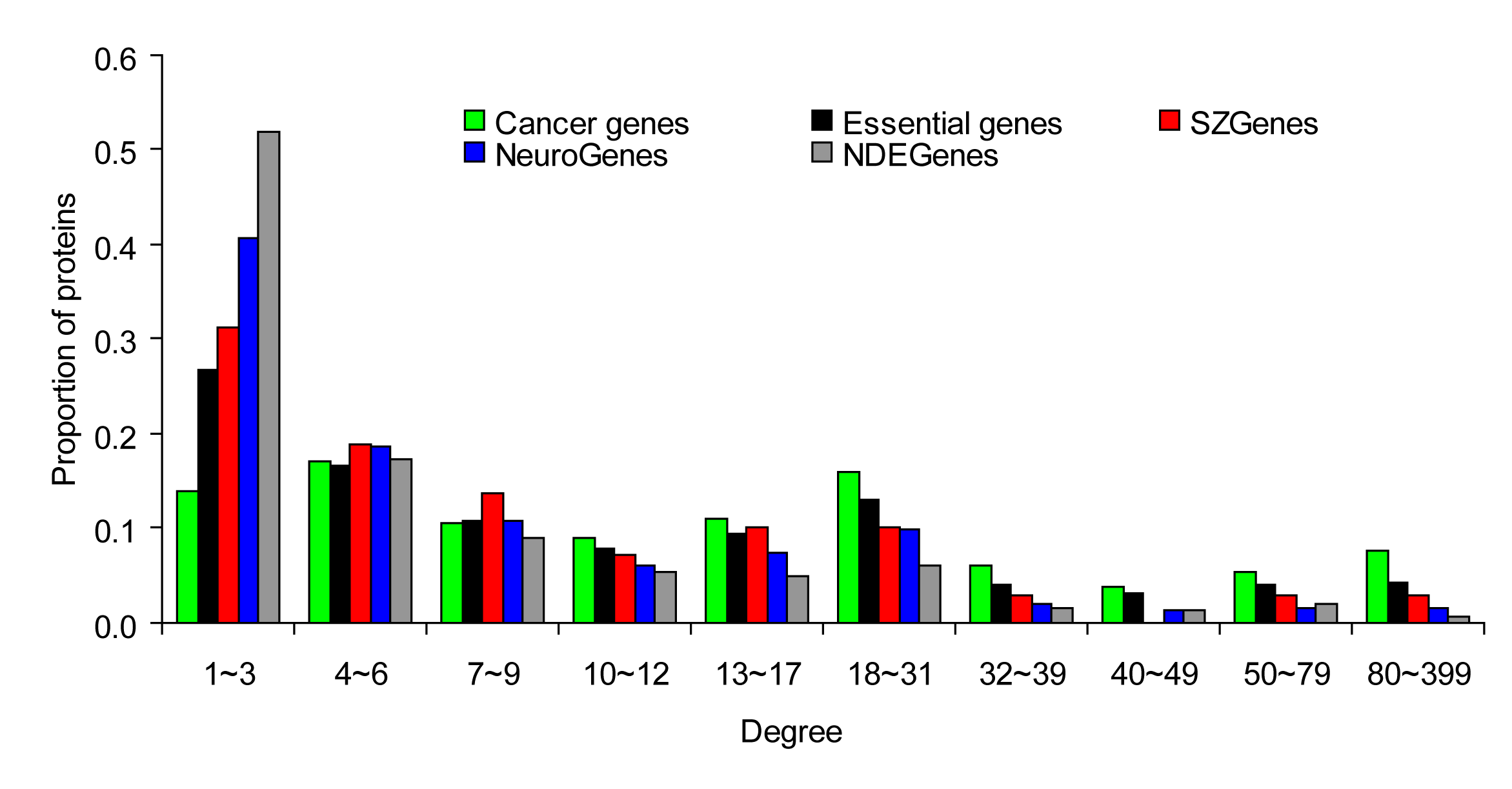

Supplement: Figure S1 — Degree distribution of five gene sets. Y-axis represents the proportion of proteins having a specific degree. (0.31 MB TIF) [file pone.0011351.s007.tif]

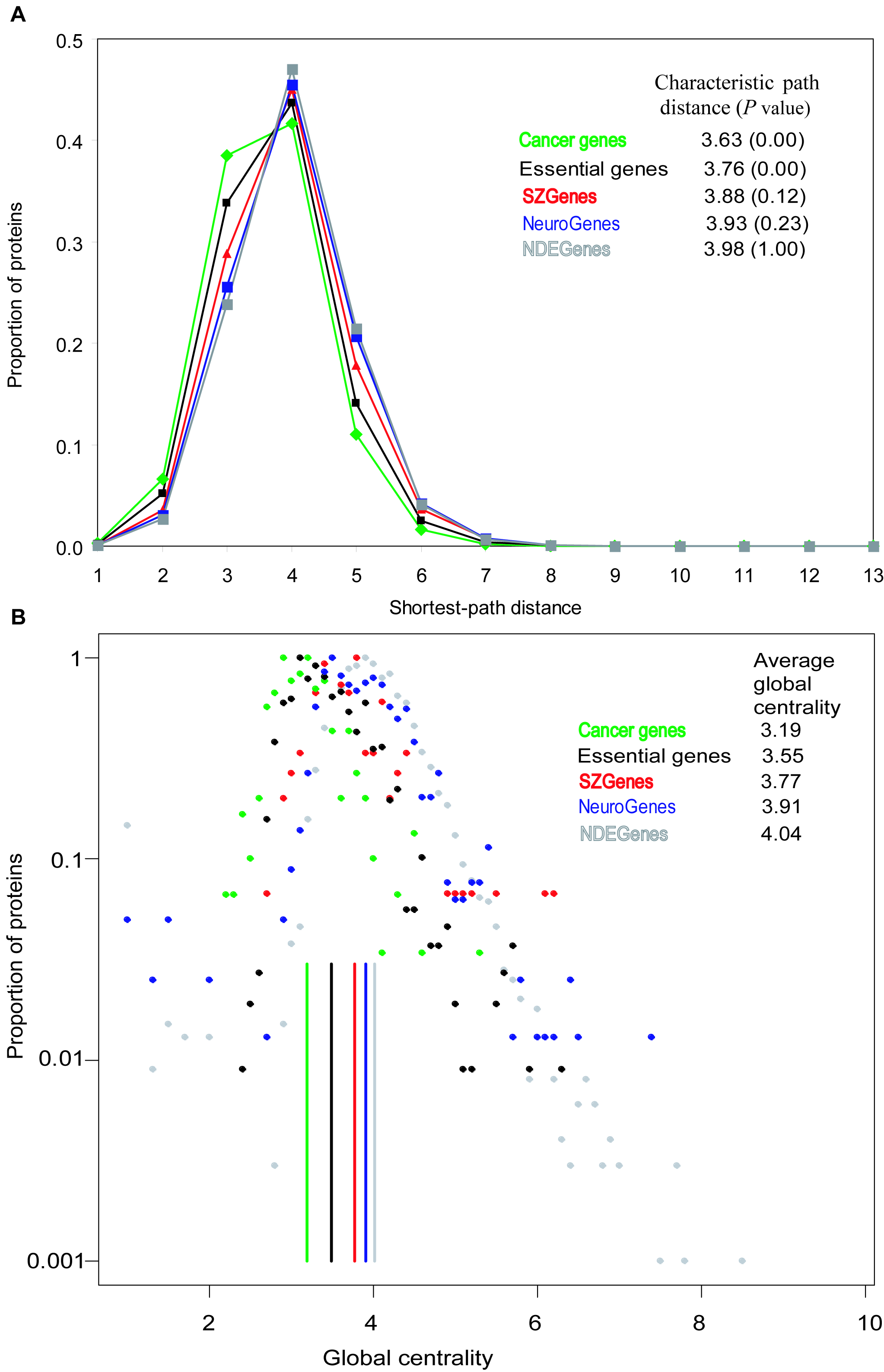

Supplement: Figure S2 — Shortest-path distance of five gene sets. (A) Characteristic shortest-path distance distribution. Y-axis is the proportion of proteins having a specific characteristic shortest-path distance. The average characteristic shortest-path distance and its empirical P value for randomness from the human interactome are shown in the table inside. (B) Global centrality distribution. Y-axis is the proportion of proteins having a specific global centrality. Vertical line represents the average global centrality of each gene set, which is also summarized in the inside of the figure. (0.92 MB TIF) [file pone.0011351.s008.tif]

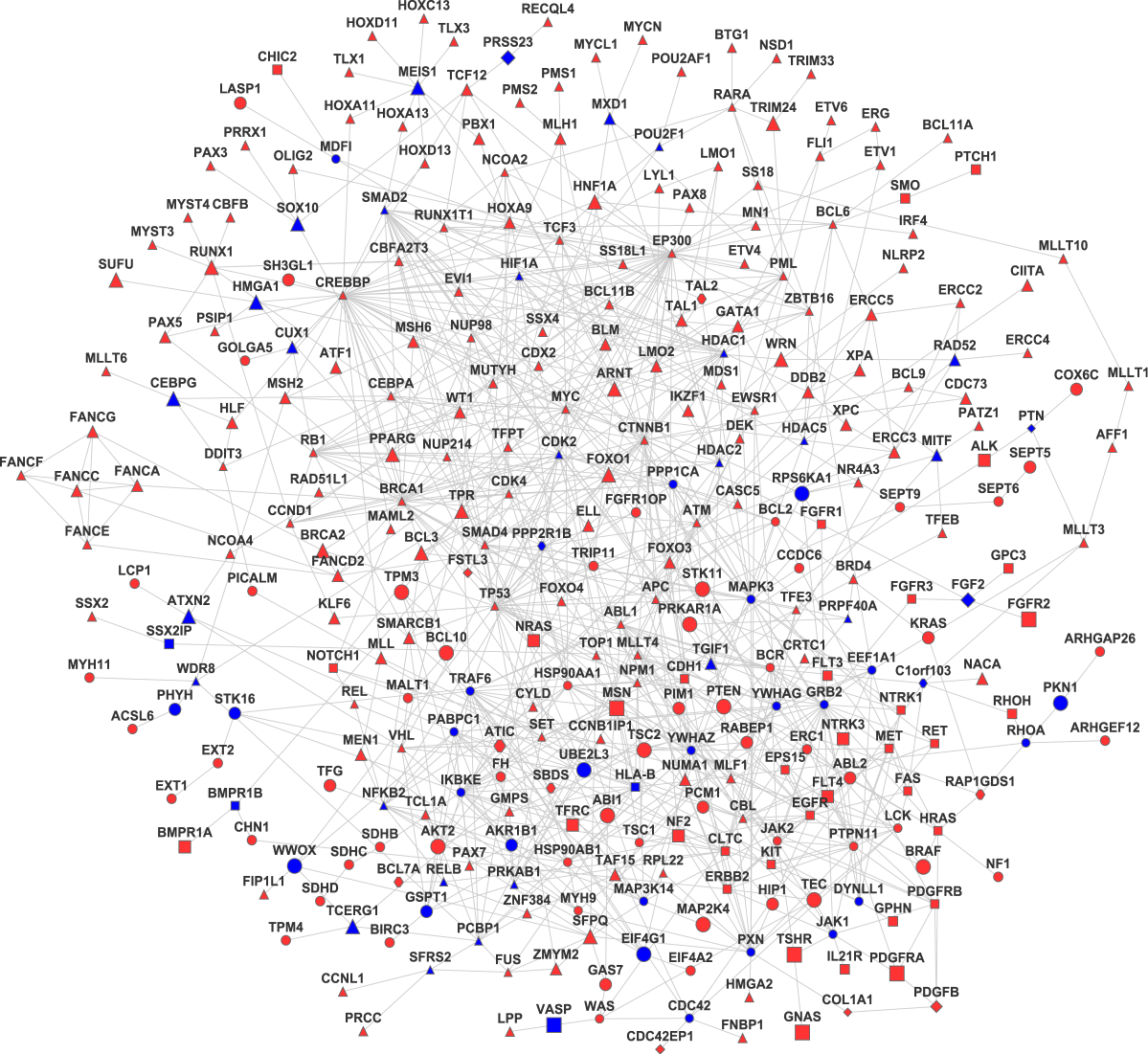

Supplement: Figure S3 — Cancer-specific network. Cancer genes are labeled in red and non-cancer genes in blue. Node area corresponds to its degree in the human interactome. Node shape indicates its cellular location: ellipse for cytoplasm, diamond for extracellular space, triangle for nucleus, square for plasma membrane, and hexagon for unknown location. (0.67 MB TIF) [file pone.0011351.s009.tif]

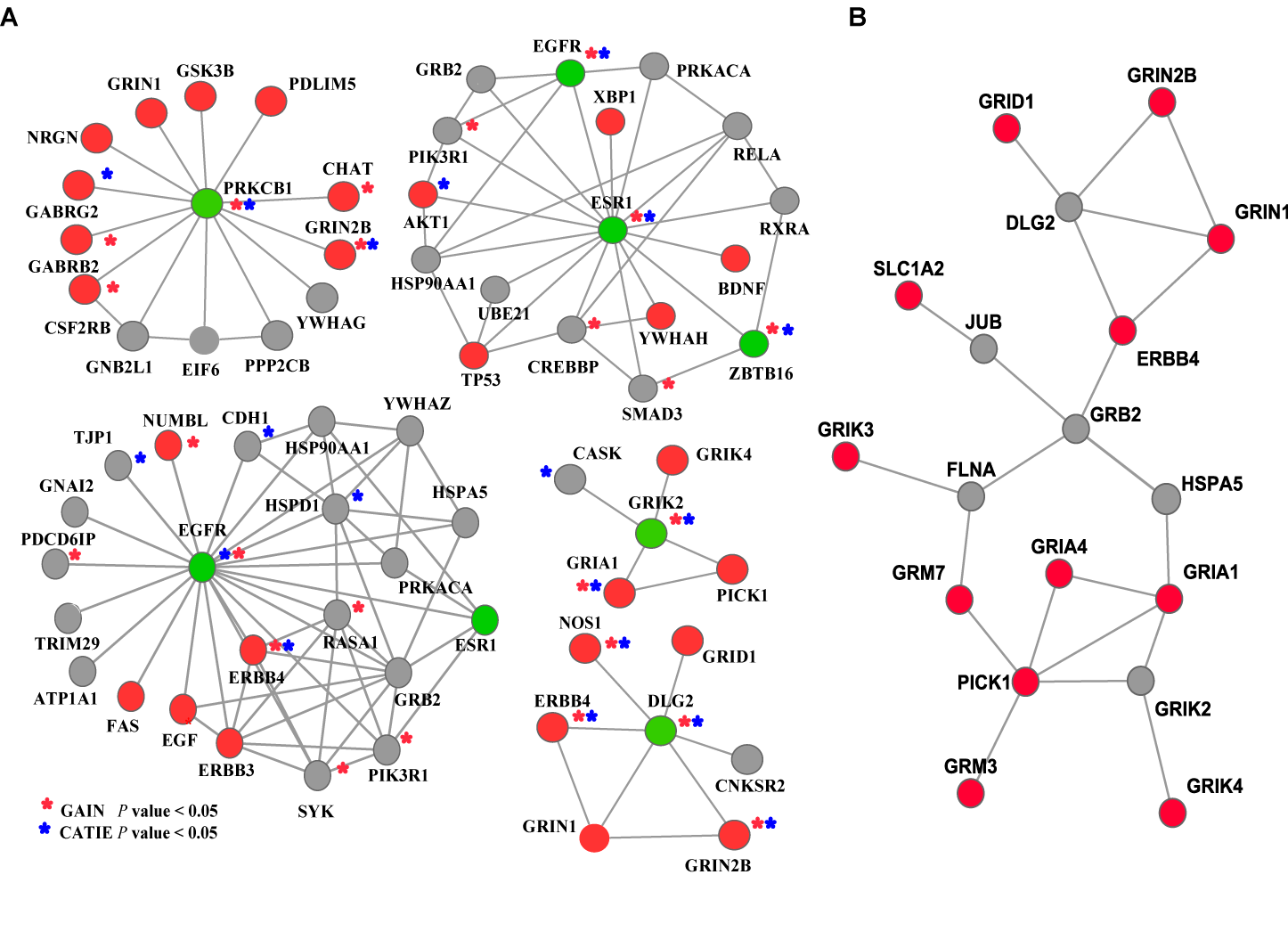

Supplement: Figure S4 — Selection of novel schizophrenia candidate genes. Nodes in red denote SZGenes and nodes in grey and green denote non-SZGenes. (A) Direct interactors of five potential schizophrenia candidate genes (in green), which are non-SZGenes but had P values <0.05 in both GAIN and CATIE GWA studies. In these subnetworks, the nodes whose genes having P value <0.05 in GAIN are labeled in red asterisk and having P value <0.05 in CATIE are labeled in blue asterisk. (B) An extracted glutamate receptor signaling subnetwork. (0.35 MB TIF) [file pone.0011351.s010.tif]
